# Involvement of central opiate receptors in modulation of centrally administered oxytocin-induced antinociception

**DOI:** 10.22038/ijbms.2018.26302.6449

**Published:** 2018-12

**Authors:** Amir Erfanparast, Esmaeal Tamaddonfard, Sahar Seyedin

**Affiliations:** 1 Division of Physiology, Department of Basic Sciences, Faculty of Veterinary Medicine, Urmia University, Urmia, Iran; 2 Faculty of Veterinary Medicine, Urmia University, Urmia, Iran

**Keywords:** Fourth ventricle, Opioid receptors, Orofacial pain, Oxytocin, Rats

## Abstract

**Objective(s):**

Oxytocin is involved in modulation of many brain-mediated functions. In the present study, we investigated the central effects of oxytocin and its receptor antagonist, atosiban on inflammatory pain. The contribution of opiate receptors was explored using non-selective and selective antagonists.

**Materials and Methods:**

The fourth ventricle of the brain of anesthetized rats was implanted with a guide cannula. Inflammatory pain in the orofacial region was induced by subcutaneous (SC) injection of formalin into the vibrissa pad, and time duration of face rubbing behavior was measured for 45 min.

**Results:**

A typical biphasic pain was observed after formalin injection. This biphasic pain behavior was attenuated by intra-fourth ventricle administration of oxytocin (12.5, 50, and 200 ng/rat). Central prior administration of 400 ng/rat atosiban (an oxytocin receptor antagonist), naloxone (a non-selective opiate receptor antagonist), naloxonazine (a selective µ-opiate receptor antagonist), and nor-binaltorphimine (a selective κ-opiate receptor antagonist), but not naltrindole (a δ-opiate receptor antagonist), prevented oxytocin-induced (200 ng/rat) antinociception. Except for naltrindole, other antagonists increased pain intensity when used alone. Above-mentioned drugs did not alter locomotor activity.

**Conclusion:**

Oxytocin, as a neuropeptide neurotransmitter, may be involved in the supraspinal modulation of inflammatory pain through µ- and κ-, but not δ-opiate receptors.

## Introduction

Hypothalamic nuclei such as paraventricular and suprachiasmatic nuclei produced neuropeptide hormone oxytocin (1). Besides well-known functions in the reproductive system (2), this neuropeptide may be involved in the modulation of anxiety, epilepsy, addiction, memory, and yawning (3-6). Pharmacological studies have suggested that oxytocin can modulate pain mechanisms at local peripheral, spinal cord, and brain sites (7-11). 

The distribution of µ-, δ-, and κ-opiate receptors has been reported in many areas of the brain (12). These receptors have a central role in pain processing (13). Pharmacological and behavioral findings have suggested that oxytocin analgesia may be associated with the endogenous opioid system. For example, after lateral cerebral ventricle co-administration of naloxone and oxytocin, the antihyperalgesic effect of oxytocin was antagonized (14).

Nociceptive information from the orofacial region is transmitted via the trigeminal nerve to the brain areas including brainstem trigeminal complex, thalamic nuclei, and cerebral cortex (15). Several authors have shown that oxytocin can modulate trigeminal pain (16, 17). Although the centrally mediated antinociception of oxytocin on paw formalin test has been reported, scholars (18, 19) have suggested some differences between spinal and trigeminal nociceptive information transmission and processing. Therefore, this study was planned to explore the effects of oxytocin and its antagonist (atosiban) on orofacial inflammatory pain after intracerebroventricular (ICV) administration. The participation of opiate receptors was evaluated using non-selective and selective opiate receptor antagonists. Clavelou *et al.* established an orofacial model of inflammatory pain in the rat (20). To study the supraspinal processing mechanisms of orofacial pain, scholars frequently used this model of inflammatory pain (21-23). The above-mentioned drugs were also used for locomotor behavior testing. 

## Materials and Methods

***Animals***

In the present study, we used male Wistar rats (250–280 g). The animals were kept in a laboratory room under controlled conditions (light on: 07:00 AM; ambient temperature: 22 ± 0.5 ^°^C). Food and water were *ad libitum*. Veterinary Ethics Committee of the Faculty of Veterinary Medicine of Urmia University approved animal use procedures. 

***Drugs***

Oxytocin, atosiban, naloxone hydrochloride, naloxonazine dihydrochloride hydrate, naltrindole hydrochloride, and nor-binaltorphimine dihydrochloride were used in this study. They were purchased from Sigma-Aldrich Chemical Co (St Louis, MO, USA). The drugs were used freshly. 

***Fourth ventricle cannulation***

To test the chemicals, a 23-gauge guide cannula was stereotaxically implanted in the fourth ventricle of the brain under ketamine (80 mg/kg) and xylazine (8 mg/kg) anesthesia. The stereotaxic coordinates for this ventricle were: -12.5 mm from the bregma, 0 mm lateral to the midline, and -7.8 mm from the top of the skull (24). The animals were allowed to recover from surgery.

***Intra-fourth ventricle administration***

Oxytocin at doses of 3.1, 12.5, 50, and 200 ng/rat, and 400 ng/rat of atosiban, naloxone, naloxonazine, nor-binaltorphimine, and naltrindole alone and before 200 ng/rat oxytocin were intracerebroventricularly (ICV) administered using a 5-μl Hamilton syringe. A constant volume of 2 µl was injected over a period of 45 sec. In separate and prior injections schedule, atosiban, naloxone, naloxonazine, nor-binaltorphimine, and naltrindole were injected 8 min, whereas oxytocin was injected 4 min before orofacial pain induction. In the present study, the used drug doses were chosen according to the literature review (22, 25) and our preliminary experiments. Due to the proximity of orofacial pain modulating centers around the fourth ventricle, we used the intra-fourth ventricle injection procedure (26-28).

***Orofacial formalin pain***

For orofacial pain induction, 50 µl of a diluted formalin solution (1.5%,) was (SC) injected into the left vibrissa pad using a 29-gauge injection needle. Face rubbing was observed through a mirror mounted at 45 ° beneath the floor of a plexiglass observation chamber (30 cm × 30 cm × 30 cm). The duration of pain behavior was recorded at 3-min blocks for 45 min (20-23). The study protocol was performed under blind conditions.

**Figure 1:**
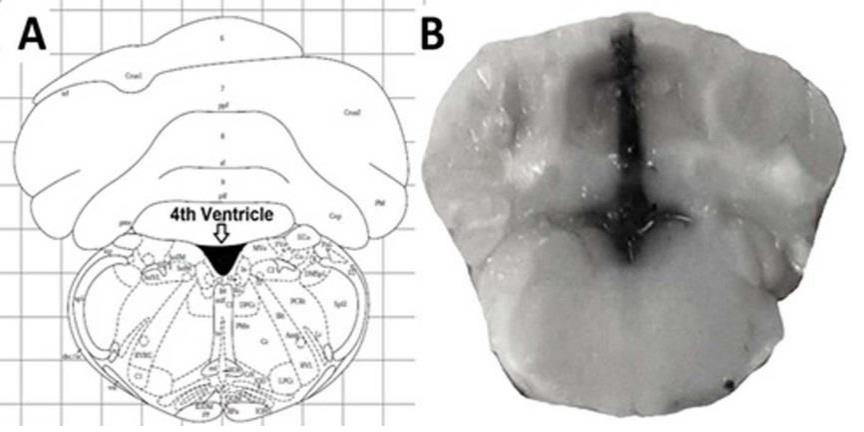
A transverse section of the rat brain showing the location of the fourth ventricle adopted from the Paxinos and Watson atlas (A). Location of the cannula placement and injection site in the fourth ventricle in the present study (B)

**Figure 2:**
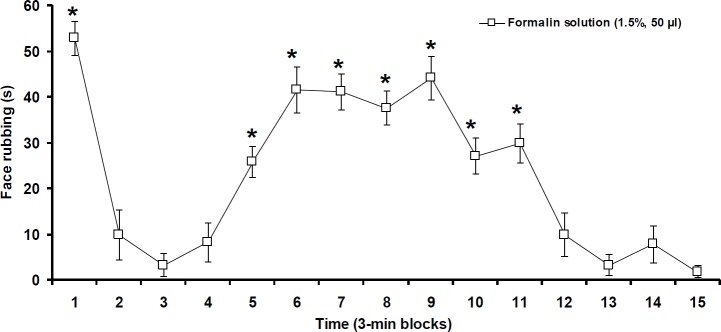
Pain behavior induced by formalin injection into the vibrissa pad. Data were shown as means±SEM. * *P*<0.05 different from other 3-min blocks

**Figure 3:**
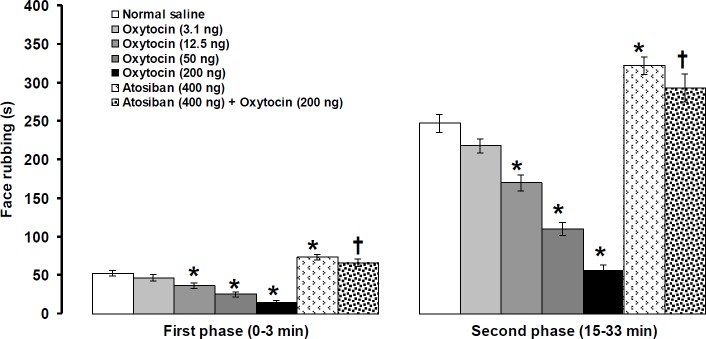
Effects of Intra-fourth ventricle injection of oxytocin, atosiban alone, and atosiban before oxytocin on formalin-induced orofacial pain. Pain behavior was recorded 4 and 8 min after central injection of oxytocin and atosiban, respectively. Data were shown as means±SEM. * *P*<0.01 different from normal saline, and † *P*<0.001 different from oxytocin (200 ng) treated groups

**Figure 4:**
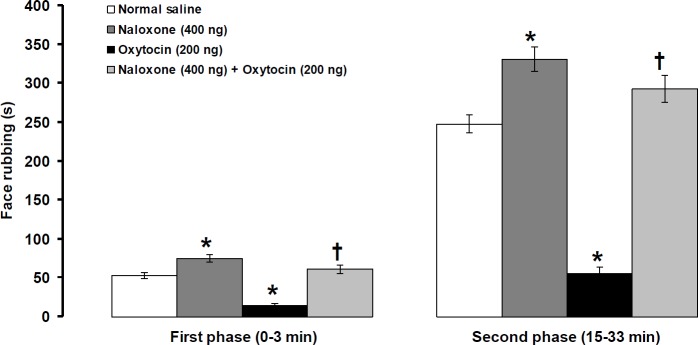
Effects of intra-fourth ventricle injection of naloxone alone and naloxone before oxytocin on formalin-induced orofacial pain. Pain behavior was assessed 4 and 8 min after central injection of oxytocin and naloxone, respectively. Data were shown as means ± SEM. * *P*<0.01 different from normal saline, and † *P*<0.001 different from oxytocin (200 ng) treated groups

**Figure 5:**
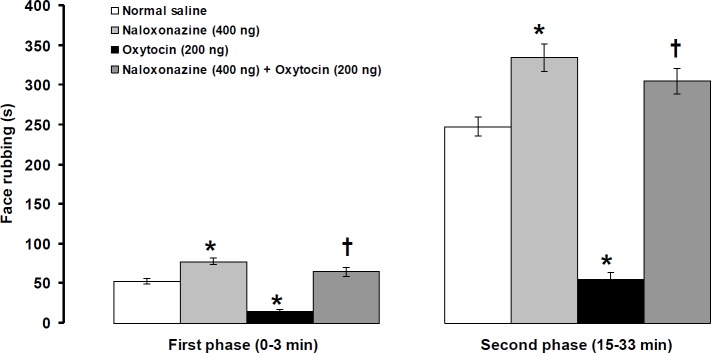
Effects of intra-fourth ventricle injection of naloxonazine alone and naloxonazine before oxytocin on formalin-induced orofacial pain. Pain behavior was assessed 4 and 8 min after central injection of oxytocin and naloxonazine, respectively. Data were shown as means ±SEM (n=6). * *P*<0.01 different from normal saline, and †* P*<0.001 different from oxytocin (200 ng) treated groups

**Figure 6:**
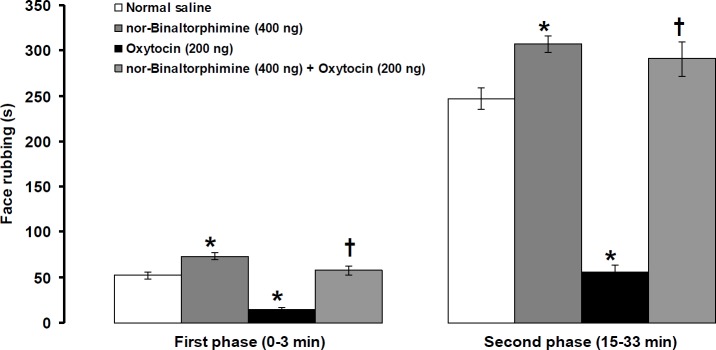
Effects of intra-fourth ventricle injection of nor-binaltorphimine alone and nor-binaltorphimine before oxytocin on formalin-induced orofacial pain. Pain behavior was assessed 4 and 8 min after central injection of oxytocin and nor-binaltorphimine, respectively. Data were shown as means±SEM. * *P*<0.01 different from normal saline and †* P*<0.001 different from oxytocin (200 ng) treated groups

**Figure 7:**
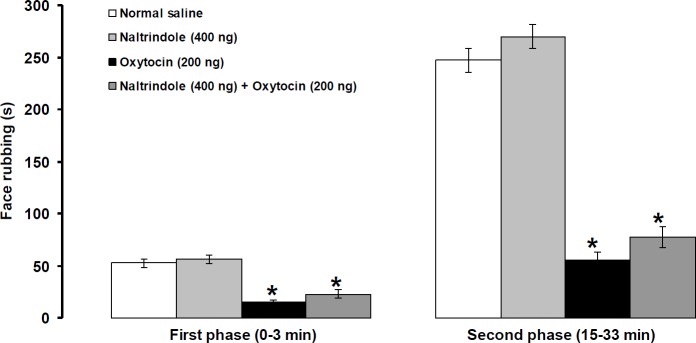
Effects of intra-fourth ventricle injection of naltrindole alone and naltrindole before oxytocin on formalin-induced orofacial pain. Pain behavior was assessed 4 and 8 min after central injection of oxytocin and naltrindole, respectively. Data were shown as means± SEM. * *P*<0.01 different from the normal saline treated group

**Figure 8:**
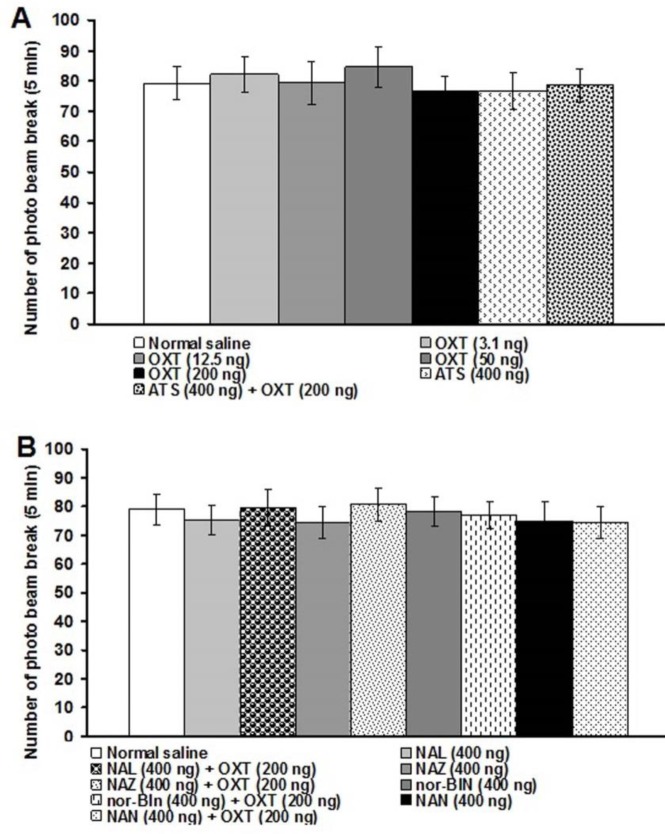
Effects of central injection of oxytocin and atosiban (A), central injection of naloxone, naloxonazine, nor-binaltorphimine and naltrindole (B) on locomotor behavior. Locomotor behavior was recorded in a 5-min session. Data were shown as means±SEM. No significant differences were seen among groups

***Locomotor activity***

An electronic activity box (BorjSanat, Tehran, Iran) was used to assess locomotor behavior. The animals were placed directly in one corner of the activity box, and the number of photobeam breaks due to movement of the animal was monitored in a 5-min session. 

***Cerebral ventricle cannula location***

After (ICV) injection of 2 µl methylene blue, animals were euthanized, the brains removed and placed in 10% formalin solution. Two days later, the brains were sectioned into 50–100 µm slices and viewed under a loupe to observe methylene blue distribution in the fourth ventricle (24). 

***Statistical analysis***

The GraphPad Prism (5.3) software (GraphPad Software, San Diego, CA, USA) was used for statistical analysis. Repeated measures analysis of variance (ANOVA) followed by Tukey’s test were employed for data obtained from SC injection of normal saline and formalin into the vibrissa pad. The effects of chemicals on pain phases and also on locomotor activity were analyzed by one-way ANOVA followed by Tukey’s test. The effects of atosiban, naloxone, naloxonazine, nor-binaltorphimine, and naltrindole used alone were analyzed using unpaired t-test. All values are expressed as the mean±SEM. Statistical significance was set at *P*<0.05.

## Results

***Cannula placement verification***

Figure 1 shows the cannula tip placement in the fourth ventricle of the brain. Figure 1A shows a schematic figure of the fourth ventricle of the brain provided from the atlas of Paxinos and Watson (24). Figure 1B shows the distribution of methylene blue in the fourth ventricle of the brain. 

***Orofacial pain behavior***

A weak pain behavior (2.17 ± 1.05 sec) was observed after (SC) injection of normal saline only at the first 3-min block (data not shown). After SC injection of formalin, the first and 5^th^–11^th^ 3-min blocks significantly (F_(14,89)_=21.301, *P*<0.05) showed more intensive pain behavior in comparison with 2^nd^–4^th^ and 12^th^–15^th^ 3-min blocks (Figure 2). 

***Effects of intra-fourth ventricular administration of oxytocin, atosiban alone, and atosiban prior to oxytocin on formalin-induced pain behavior***

Central injection of oxytocin at a dose of 3.1 ng/rat was without effect, whereas at doses of 12.5, 50, and 200 ng/rat, oxytocin significantly attenuated both the first (F_(4,29)_=20.891, *P*<0.01, Figure 3) and second (F_(4,29)_=67.194, *P*<0.01, Figure 3) phases of pain behavior. In the first (df=10, t= 4.018, *P*<0.01, Figure 3) as well as the second (df=10, t= 4.206, *P*<0.01, Figure 3) phases intensity of pain was increased after central injection of atosiban (400 ng/rat). Prior central injection of atosiban (400 ng/rat) significantly inhibited the antinociceptive effects of oxytocin (200 ng/rat) on the first (F_(2,17)_=67.0128, *P*<0.001, Figure 3) and second (F_(2,17)_=123.601, *P*<0.001, Figure 3) phases of pain. 

***Effects of intra-fourth ventricular injection of naloxone alone and prior to oxytocin on pain behavior induced by formalin***

Naloxone alone (400 ng/rat) significantly increased the first (df=10, t= 3.682, *P*<0.01, Figure 4) and second (df=10, t= 4.265, *P*<0.01, Figure 4) phases of pain intensity. The suppressive effects of oxytocin (200 ng/rat) on the first (F_(2,17)_=50.651, *P*<0.001, Figure 4) and second (F_(2,17)_=110.903, *P*<0.001, Figure 4) phases of pain were prevented by prior central injection of naloxone (400 ng/rat). 

***Effects of intra-fourth ventricular injection of naloxonazine alone and prior to oxytocin on pain behavior induced by formalin***

Intra-fourth ventricle injection of naloxonazine (400 ng/rat) significantly increased the intensity of pain at the first (df=10, t= 4.151, *P*<0.01, Figure 5) and second (df=10, t= 4.226, *P*<0.01, Figure 5) phases. Naloxonazine (400 ng/rat) significantly prevented suppressive effects of oxytocin (200 ng/rat) on both the first (F_(2,17)_=58.765, *P*<0.001, Figure 5) and second (F_(2,17)_=114.304, *P*<0.001, Figure 5) phases of pain when used before oxytocin.

***Effects of intra-fourth ventricular injection of nor-binaltorphimine alone and prior to oxytocin on pain behavior induced by formalin***

The first phase (df=10, t= 3.887, *P*<0.01, Figure 6) as well as the second phase (df=10, t=4.028, *P*<0.01, Figure 6) of pain intensity significantly increased after central administration of nor-binaltorphimine (400 ng/rat). The suppressive effects of oxytocin (200 ng/rat) on the first (F_(2,17)_=60.18, *P*<0.001, Figure 6) and second (F_(2,17)_=120.7, *P*<0.001, Figure 6) phases of pain were inhibited by prior administration of 400 ng/rat nor-binaltorphimine. 

***Effects of intra-fourth ventricular injection of naltrindole alone and prior to oxytocin on pain behavior induced by formalin***

Central injection of nantrindole (400 ng/rat) not only had no effect on pain intensity but also did not prevent oxytocin (200 ng/rat)-induced antinociceptive effects (Figure 7). 

***Effects of central administration of oxytocin, atosiban, and opioid receptor antagonists on locomotor activity***

The photobeam break number after central injection of normal saline was 79.2±5.38 in a 5-min session. None of the above-used chemicals changed the number of photobeam break (Figure 8).

## Discussion

Many scholars have reported a biphasic pain behavior (face rubbing) after SC injection of diluted formalin solutions (1%, 1.5%, and 2%) into lip and vibrissa pad (20, 22, 23, 29). Direct stimulation of C-nociceptors reflects the first phase, whereas integration among nociceptors and spinal and brainstem signaling may associate with the second phase (30). Although other behaviors including nose grooming, face scratching due to SC injection of formalin into the orofacial region have been reported (31, 32), face rubbing has been accounted as a specific pain behavior resulting from vibrissa pad injection of formalin (20, 23). A typical biphasic pain behavior obtained in our present study confirms the other findings (20, 23, 29). 

In the rat brain, the amygdala (AMY), the hippocampus (HIP), the nucleus accumbens (NAc), the ventral tegmental area (VTA), the periaqueductal gray (PAG), the rostral ventrolateral medulla (RVLM), and the spinal cord receive projections from oxytocin neurons (33-35). In addition, oxytocin receptors are expressed in parallel with oxytocin axons in the central nervous system (33, 34). Only one selective receptor for oxytocin has been characterized although oxytocin can act via vasopressin receptors at high concentrations (36). This receptor is coupled to phospholipase C via G_αq11_ protein activation, controlling inositol triphosphate and diacylglycerol generation leading to liberation of calcium ions from intracellular stores and activation of protein kinase C, respectively (33). Oxytocin modulates pain at the central nervous system level. Intrathecal or (ICV) injection oxytocin and anti-oxytocin serum increased and decreased nociception threshold, respectively (25). In addition, oxytocin concentration in the caudate nucleus (CdN) and PAG was increased after noxious stimulation (37, 38). To date, the central effect of oxytocin on formalin-induced orofacial pain was not investigated. Zubrzycka *et al.* (16), showed that ICV injection of oxytocin suppressed the tongue movements induced by tooth pulp stimulation, and prior central administration of atosiban blocked this effect (16). In addition, ICV injection of oxytocin (100-600 ng/rat) attenuated mechanical hypersensitivity following hind paw incision (39). In this context, an antihyperalgesic effect of centrally administered oxytocin has been reported after mechanical and thermal stimulation of carrageenan-injected paw in mice (40). The results of the present study reveal for the first time that central oxytocin through its receptor can modulate the orofacial pain induced by formalin.

Our results showed the involvement of central µ- and κ-, but not δ-opioid receptor in the processing of orofacial pain. Scholars reported moderate to high densities of µ**-** and κ**-**opioid receptor and a moderate density of delta opioid receptor in ascending pain modulation centers such as raphe nucleus, locus coeruleus and parabrachial nucleus (12). In addition, tooth pulp stimulation induced opioid receptors expression with high mu opioid receptor expression in brainstem structures such as periaqueductal grey (41). Using central injection of opioid receptor selective antagonists, it was reported that microinjection of naloxone, CTOP (Cys2, Try3, Orn5, Pen7amide, a mu-opioid receptor antagonist) and nor-binaltorphimine into the subnucleus caudalis of the spinal trigeminal nucleus increased temporomandibular injected formalin-induced nociceptive response (42). In addition, the inflammatory pain of temporomandibular region increased spinal trigeminal subnucleus caudalis level of β-endorphins (43). It seems that central µ- and κ-opioid receptors may have a potent role in the modulation of inflammatory pain originating from the orofacial region. 

Naloxone is a competitive antagonist of µ- and κ-opiate receptors with higher affinity for µ- receptors (44, 45), and is frequently used to explore the contribution of endogenous opioid and non-opioid systems in central modulation of orofacial pain (15, 22, 23). Prior ICV injection of naloxone prevented the antihyperalgesic effects of centrally-administered oxytocin in the carrageenan model of inflammation and hyperalgesia in mice (40). To clarify the contribution of opiate receptors in oxytocin-induced antinociception, we used selective mu-, kappa-, and delta-opioid receptor antagonists, before oxytocin. In addition to a direct effect, many scholars suggested an indirect effect of oxytocin on pain modulation, which is mediated through opioid receptors. Beta-funaltrexamine (a mu-opioid receptor antagonist) and nor-binaltorphimine, but not by naltrindole attenuated centrally administered oxytocin-induced antinociception (46). Moreover, the concentrations of endogenous opioid peptides such as leucine-enkephalin and methionine-enkephalin were increased after microinjection of oxytocin into the PAG (47). According to our present results, a central interaction between opioid and oxytocin receptors in the modulation of analgesia in the orofacial inflammatory pain is indicated.

Our present results showed that locomotor activity has not been influenced by the used chemicals. Although there are no reports showing the sedation or hyperactivity of the above-mentioned drugs, Peterson *et al.* (48) reported an increase in the amount of locomotor activity as well as antinociceptive effect after chronic SC injection of oxytocin in ovariectomized rats. Therefore, centrally administered oxytocin-induced antinociception observed in our present study could be due to its effect on pain modulating centers. 

## Conclusion

Our present findings showed that ICV injection of oxytocin reduced neurogenic and inflammatory pain originating from the orofacial region. Oxytocin receptors may be involved in this effect. Naloxone inhibited oxytocin-induced antinociception. Moreover, naloxonazine and norbinaltorphimine, but not naltrindole, prevented centrally-administered oxytocin-induced antinociceptive effects.

## References

[1] Tom N, Assinder SJ (2010). Oxytocin in health and disease. Int J Biochem Cell Biol.

[2] Veening GJ, de JongTR, WaldingerMD, Korte SM, Olivier B (2015). The role of oxytocin in male and female reproductive behavior. Eur J Pharmacol.

[3] CollinsGT, EquibarJR (2010). Neuropharmacology of yawning. Front Neurol Neurosci.

[4] MacDonald K, Feifel D (2014). Oxytocinʼs role in anxiety: a critical appraisal. Brain Res.

[5] Sarnyai Z, Kovacs GL (2014). Oxytocin in learning and addiction: from early discoveries to the present. Pharmacol Biochem Behav.

[6] Erfanparast A, Tamaddonfard E, Henareh-Chareh F (2017). Intra-hippocampal microinjection of oxytocin produced antiepileptic effect on the pentylenetetrazol-induced epilepsy in rats. Pharmacol Rep.

[7] Engle MP, Ness TJ, Robbins MT (2012). Intrathecal oxytocin inhibits visceromotor reflex and spinal neuronal responses to noxious distention of the rat urinary bladder. Reg Anesth Pain Med.

[8] Gonzalez-Hernandez A, Rajos-Piloni G, Condes-Lara M (2014). Oxytocin and analgesia: future trends. Trends Pharmacol Sci.

[9] Rash JA, Aguire-Camacho A, Campbell TS (2014). Oxytocin and pain-a systematic review and synthesis of findings. Clin J Pain.

[10] Abbasnezhad A, Khazdir MR, Kianmehr M (2016). The role of nitric oxide on the oxytocin induced analgesia in mice. Iran J Basic Med Sci.

[11] Xin Q, Bai B, LiuW (2017). The analgesic effects of oxytocin in the peripheral and central nervous system. Neurochem Int.

[12] Mansour A, Fox CA, Burke S, Meng F, Thompson RC, Akil H (1994). Mu, delta, kappa opioid receptor mRNA expression in the rat CNS: an in situ hybridization study. J Comp Neurol.

[13] Bodnar RJ (2016). Endogenous opiates and behavior 2015. Peptides.

[14] Russo R, DʼAgostino G, MattaceRaso G, Avagliano C, Cristiano C, Meli R (2012). Central administration of oxytocin reduces hyperalgesia in mice: implication for cannabinoid and opioid systems. Peptides.

[15] Sessle BJ (2011). Peripheral and central mechanisms of orofacial inflammatory pain. Int Rev Neurobiol.

[16] Zubrzycka M, Jakub F, Janecka A (2005). Inhibition of trigemino-hypoglossal reflex in rats by oxytocin is mediated by µ and κ opioid receptors. Brain Res.

[17] Wang YL, Yuan Y, Yang J, Wang CH, Pan YJ, Lu L (2013). The interaction between the oxytocin and pain modulation in headache patients. Neuropeptides.

[18] Nones CF, Reis RC, Jesus CH, Veronez DA, Cunha JM, Chichorro JG (2013). Orofacial sensory changes after streptozotocin-induced diabetes in rats. Brain Res.

[19] Fried K, Bongenhielm U, Boissonade FM, Robinson PP (2001). Nerve injury-induced pain in the trigeminal system. Neuroscientist.

[20] Clavelou P, Pajot J, Dallel R, Raboisson P (1989). Application of the formalin test to the study of orofacial pain in the rat. Neurosci lett.

[21] Duale C, Sierralta F, Dallel R (2007). Analgesia induced by morphine microinjected into the nucleus raphe magnus: effect on tonic pain. Curr Drug Deliv.

[22] Erfanparast A, Tamaddonfard E, Nemati S (2017). Effects of intra-hippocampal microinjection of vitamin B12 on the orofacial pain and memory impairments induced by scopolamine and orofacial pain in rats. Physiol Behav.

[23] Tamaddonfard E, Erfanparast A, Farshid AA, Khalilzadeh E (2011). Interaction between histamine and morphine at the level of the hippocampus in the formalin-induced orofacial pain in rats. Pharmacol Rep.

[24] Paxinos G, Watson C (1997). The rat brain in stereotaxic coordinates.

[25] Yang J, Yang Y, Chen JM, Liu WY, Wang CH, Lin BC (2007). Central oxytocin enhances antinociception in the rat. Peptides.

[26] Bourne S, Machada AG, Nagel SJ (2014). Basic anatomy and physiology of pain pathways. Neurosurg Clin N Am.

[27] Ossipov MH, Dussor GO, PorrecaF (2010). Central modulation of pain. J Clin Invest.

[28] Vanegas H, Schaible HG (2004). Descending control of persistent pain: inhibitory or facilitatory. Brain Res Rev.

[29] Donatti AF, Araujo RM, Soriano RN, Azevedo LU, Leite-Panissi CA, Branco LG (2014). Role of hydrogen sulfide in the formalin-induced orofacial pain in rats. Eur J Pharmacol.

[30] Dallel R, Raboisson P, Clavelou P, Saade M, Woda A (1995). Evidence for a peripheral origin of the tonic nociceptive response to subcutaneous formalin. Pain.

[31] Ahn DK, Lee KR, Lee HJ, Kim SK, Choi HS, Lim EJ (2005). Intracisternal administration of chemokines facilitated formalin-induced behavioral response in the orofacial area of freely moving rats. Brain Res Bull.

[32] Yang GY, Woo YW, Park MK, Bae YC, Ahn DK, Bonfa E (2010). Intracisternal administration of NR2 antagonists attenuates facial formalin-induced nociceptive behavior in rats. J Orofacl Pain.

[33] Gimpl G, Fahrenholz F (2001). The oxytocin receptor system: structure, function, and regulation. Physiol Rev.

[34] Grinevich V, Knobloch-Bollmann HS, Eliava M, Chini B (2016). Assembling the puzzle: pathways of oxytocin signaling in the brain. Biol Psychiatry.

[35] Lee SK, Ryu PD, Lee SY (2013). Differential distributions of neuropeptides in hypothalamic paraventricular nucleus neurons projecting to the rostral ventrolateral medulla in the rat. Neurosci Lett.

[36] Manning M, Misicka A, Olma A, Bankowski K, Stoev S, Chini B (2012). Oxytocin and vasopressin agonists and antagonists as research tools and potential therapeutics. J Neuroendocrinol.

[37] Yang J, Pan YJ, Zhao Y, Qiu PY, Lu L, Li P (2011). Oxytocin in the rat caudate nucleus influences pain modulation. Peptides.

[38] Yang J, Li P, Liang JY, Pan YJ, Yan XQ, Yan FL (2011). Oxytocin in the periaqueductal grey regulates nociception in the rat. Regul Pept.

[39] Zhang Y, Yang Y, Dai R, Wu H, Li C, Guo Q (2015). Oxytocin in the paraventricular nucleus attenuates incision-induced mechanical allodynia. Exp Ther Med.

[40] Russo R, D’Agostino G, Mattace Raso G, Avagliano C, Cristiano C, Meli R (2012). Central administration of oxytocin reduces hyperalgesia in mice: implication for cannabinoid and opioid systems. Peptides.

[41] Zubrzycka M1, Szemraj J, Janecka A (2011). Effect of tooth pulp and periaqueductal central gray stimulation on the expression of genes encoding the selected neuropeptides and opioid receptors in the mesencephalon, hypothalamus and thalamus in rats. Brain Res.

[42] Macedo CG, Fanton LE, Fischer L, Tambeli CH (2016). Coactivation of μ- and κ-opioid receptors may mediate the protective effect of testosterone on the development of temporomandibular joint nociception in male rats. J Oral Facial Pain Headache.

[43] Araújo IW, Chaves HV, Pachêco JM, Val DR, Vieira LV, Santos R (2017). Role of central opioid on the antinociceptive effect of sulfated polysaccharide from the red seaweed Solieria filiformis in induced temporomandibular joint pain. Int Immunopharmacol.

[44] Helm S, Trescot AM, Colson J, Sehgal N, Silverman S (2008). Opioid antagonists, partial agonists, and agonists/antagonists: the role of office-based detoxification. Pain Physician.

[45] Trescot AM, Datta S, Lee M, Hansen H (2008). Opioid pharmacology. Pain Physician.

[46] Gao L, Yu LC (2004). Involvement of opioid receptors in the oxytocin-induced antinociception in the central nervous system of rats. Regul Pept.

[47] Yang J, Liang JY, Li P, Pan YJ, Qiu PY, Zhang J (2011). Oxytocin in the periaqueductal gray participates in pain modulation in the rat by influencing endogenous opiate peptides. Peptides.

[48] Peterson M, Eklund M, Uvnas-Moberg K (2005). Oxytocin decreases corticosterone and nociception and increases motor activity in OVX rats. Maturitas.

